# Application of stimuli-responsive hydrogel in brain disease treatment

**DOI:** 10.3389/fbioe.2024.1450267

**Published:** 2024-07-18

**Authors:** Bingqing Xie, Huangfan Xie

**Affiliations:** ^1^ Laboratory of Neurological Diseases and Brain Function, The Affiliated Hospital, Southwest Medical University, Luzhou, Sichuan, China; ^2^ Institute of Epigenetics and Brain Science, Southwest Medical University, Luzhou, Sichuan, China

**Keywords:** stimuli-responsive, hydrogels, brain disease, drug delivery, tissue engineering

## Abstract

Treating brain diseases presents significant challenges due to neuronal degeneration, inflammation, and the intricate nature of the brain. Stimuli-responsive hydrogels, designed to closely resemble the brain’s extracellular matrix, have emerged as promising candidates for controlled drug delivery and tissue engineering. These hydrogels have the unique ability to encapsulate therapeutic agents and release them in a controlled manner when triggered by environmental stimuli. This property makes them particularly suitable for delivering drugs precisely to targeted areas of the brain, while minimizing collateral damage to healthy tissue. Their preclinical success in treating various brain diseases in animal studies underscores their translational potential for human brain disease treatment. However, a deeper understanding of their long-term behavior, biodistribution, and biocompatibility within the brain remains crucial. Furthermore, exploring novel hydrogel systems and therapeutic combinations is paramount for advancing towards more effective treatments. This review summarizes the latest advancements in this field over the past 5 years, specifically highlighting preclinical progress with novel stimuli-responsive hydrogels for treating brain diseases.

## 1 Introduction

Brain diseases are one of the primary neurological disorders with high incidence, including traumatic brain injury (TBI), Alzheimer’s disease, Parkinson’s disease, dementia, epilepsy, schizophrenia, stroke, depression, and glioblastoma (GBM) (Feigin et al., 2020). While their etiologies vary, they primarily involve progressive neuronal degeneration and inflammation. In most cases, the treatments to brain disease are limited to symptom reduction and palliative care. Curative therapies that can reverse the illness are lacking. Main challenges for brain disease treatment lie in the lack of structural support allowing repopulation of brain tissue from cell loss and the barriers for efficient drug delivery and release caused by blood-brain barriers (BBB) or other biological environment in brain tissues (Terstappen et al., 2021).

Stimuli-responsive hydrogels, characterized by their three-dimensional cross-linked polymer structure, possess unique properties that render them suitable for addressing various challenges in brain disease treatment. These hydrogels dynamically adapt their characteristics, including mechanical properties, swelling capacity, hydrophilicity, and permeability to bioactive molecules, in response to environmental stimuli such as temperature, pH, and biological agents (Mantha et al., 2019). Their water-absorbing and swelling characteristics mimic the natural extracellular matrix (ECM), fostering an optimal environment for cellular growth and tissue engineering (Ma and Huang, 2020). Furthermore, they excel in encapsulating a range of therapeutic agents, including neuroprotective agents, chemotherapeutic drugs and even cells, and releasing them in a controlled manner, positioning them as exceptional candidates for drug delivery systems (Ghosh et al., 2024). Stimuli-responsive hydrogels have demonstrated encouraging potential in treating brain diseases over the past few decades (Peressotti et al., 2021; Grimaudo et al., 2022). However, further understanding of cell-biomaterial interaction and developing safer, more effective hydrogel systems are crucial for their translation into human therapy.

This review covers the last 5 years of fundamental research on stimuli-responsive hydrogels, discussing their characteristics, design, and preclinical applications in drug delivery and tissue engineering for brain disease treatment. It also highlights challenges and opportunities for future research in this field.

## 2 Characteristics of stimuli-responsive hydrogels

### 2.1 General characteristics of hydrogels

Based on polymer source, hydrogels are classified as natural, synthetic, or semi-synthetic. Natural hydrogels have biocompatibility and biodegradability but limited stability and mechanical strength (Alawami and Tannous, 2021; Janas-Naze and Zhang, 2022). Synthetic hydrogels provide mechanical strength but lack biological activity (Zhang and Huang, 2020). Semi-synthetic hydrogels combine the best of both, improving bioactivity and tunable properties (Dienes et al., 2021).

Hydrogel fabrication involves polymerization and crosslinking to create networks with varying mechanical and swelling properties (Grimaudo et al., 2022; Mashabela et al., 2022). Hydrogels can be administered intravenously, intracerebrally, intratumorally, or intranasally to treat brain diseases (Mashabela et al., 2022). Intravenous injection faces BBB penetration challenges, while intracerebral/intratumoral delivery demands hydrogels with high biocompatibility and safety. Intranasal administration offers direct access to the brain, bypassing the BBB, but is limited by nasal cavity size, mucociliary clearance, enzymatic degradation, and potential drug-induced irritation/neurotoxicity (Cassano et al., 2021).

### 2.2 Classification of stimuli-responsive of hydrogels

Stimuli-responsive hydrogels are categorized as physical, chemical, or biological-responsive, depending on their triggering factors. Here we summarize the characteristics of various stimuli-responsive hydrogels suitable for brain disease treatment.

#### 2.2.1 Physical-responsive hydrogels

Physical-responsive hydrogels can be classified into thermo-, photo-, electro-, magnetic-, ultrasound-responsive types, each sensitive to temperature, light, electrical stimulation, magnetism and ultrasound respectively. Among these, thermo-, photo-, and electro-responsive hydrogels are the most widely used due to their practicality and effectiveness.

Thermo-responsive hydrogels shrink or expand with temperature changes, featuring hydrophobic (such as methyl, ethyl, and propyl) and hydrophilic groups (like amide and carboxyl) (Tang et al., 2021). Poloxamers (e.g., P407 and P188), also called Pluronics^®^, are commonly used for intranasal drug delivery due to their mucoadhesive and sol-gel transition properties (Ahmed et al., 2019; Abdeltawab et al., 2020). Hydrogels made from these poloxamers effectively deliver drugs like tetrandrine and rotigotine to the brain, but they lack mechanical strength and viscosity in physiological conditions (Wang et al., 2020; Zhang et al., 2020). To overcome this, they are often combined with other polymers like Carbopol, chitosan, and cellulose derivatives (Luo et al., 2023). Other agents, such as gellan and xanthan gums, are also used for brain drug delivery but may be costly (Rajput et al., 2018).

Photo-responsive hydrogels have photoreceptive moieties that can capture and convert light signal to chemical signals via photoreactions like isomerization, cleavage, and dimerization, thereby changing hydrogel’s physical and chemical properties (Li et al., 2019). However, the primary use of ultraviolet-reactive groups in these systems restricts their biomedical applications.

Electro-responsive hydrogels, containing conducting materials, reversibly absorb and expel water upon electrical stimulation. Their hydration, flexibility, biocompatibility, and electrochemical properties make them suitable for brain implantation, enhancing neural signal transmission and ion transport (Khan et al., 2022). Integrating electroconductive materials into hydrogels reduces inflammation and material loss risks after brain implantation (Guo and Ma, 2018).

Magnetic-responsive hydrogels use magnetic particles, including γ-Fe_2_O_3_, Fe_3_O_4_ and CoFe_2_O_4_, to deliver drugs to specific sites in response to magnetic field (Zhang et al., 2023a). Currently, these hydrogels are limited to *in vitro* use due to toxicity and reproducibility concerns.

Ultrasound-responsive hydrogels, with imaging-compatible polymers or contrast agents, enhance ultrasound diagnostic accuracy. These hydrogels respond to ultrasound through three mechanisms: crosslinking disruption, hyperthermia, and acoustic streaming (Zhou et al., 2022). However, their use is currently limited to *in vitro* neural tissue engineering for structural guidance (Cheng et al., 2020).

Ion strength-responsive hydrogels alter in response to solution ion changes, resulting in protonation or deprotonation. Controlling ionic strength can regulate hydrogel reversibility for drug release. Deacylated gellan gum has been effectively utilized in the preparation process of this hydrogel (Chen et al., 2020).

#### 2.2.2 Chemical-responsive hydrogels

Chemical-responsive hydrogels are classified into pH-, ROS-, hypoxia-responsive types, each sensitive to pH, reactive oxygen species (ROS), and hypoxia respectively.

pH-responsive hydrogels are composed of polymers with hydrophobic moieties that can swell in water depending on pH (Tulain et al., 2018). Recently, hydrogels based on Schiff base chemistry have received attention due to their quick formation via aldehyde-amine bonds and reversible pH-responsive properties (Zhang et al., 2023b).

ROS-responsive hydrogels interact with ROS using oxygen-sensitive groups, which alter the hydrogel network (Gao and Xiong, 2021). Boronic acid crosslinking is used to create various ROS-responsive hydrogels from polymers like sodium alginate, hyaluronic acid, cellulose, chitosan, gelatin, etc.

Hypoxia-responsive hydrogels can be synthesized through incorporation of hypoxia-sensitive moieties. These moieties, including 2-nitro imidazole, azobenzene derivatives, and nitro benzyl derivatives, enable the hydrogels to specifically release drugs within hypoxic environment (Peng et al., 2021).

#### 2.2.3 Biological-responsive hydrogels

Biological-responsive hydrogels are classified into enzyme- and glucose-responsive types, each sensitive to enzymes or glucose.

Enzyme-responsive hydrogels incorporate biomolecules that can be cleaved by specific enzymes such as matrix metalloproteinases (MMPs), proteinase K, hyaluronidase and esterase, resulting in altered swelling properties of the gel (Sobczak, 2022). Hyaluronic acid hydrogels can be degraded by hyaluronidase and esterase, while in some cases, by adding MMP-cleavable (PLGL, GCDSGGRMSMPVSDGG) or inactive peptides (GCRDFGAIGQDGDRGG), hydrogels can be made responsive to MMPs (Jian et al., 2018; Adak et al., 2020).

Glucose-responsive hydrogels change their sol-gel behavior based on glucose levels, of which common types include concanavalin A, glucose oxidase, and phenylboronic acid (PBA) hydrogels (Morariu, 2023).

#### 2.2.4 Multiple-stimuli-responsive hydrogels

To broaden the capabilities and uses of hydrogels, there’s been a surge of interest in creating dual or even multiple-stimuli-responsive hydrogels. A straightforward method for creating these hydrogels is by incorporating multiple stimuli-responsive materials into existing composite hydrogel systems. Dual ROS/enzyme-, ROS/glucose-, pH/thermo-, ion strength/thermo-, and even triple ROS/pH/thermo-responsive hydrogels have been utilized in brain disease treatment (Table 1).

**TABLE 1:** Summary of preclinical *in vivo* study evaluating the therapeutic efficacy of stimuli-responsive hydrogels in brain disease treatment.

Class of hydrogels		Main composition/biomaterial	Crosslinker	Stimuli/encapsulates	Disease	*In vivo* models	Route of administration	Outcome	References
Thermo-responsive		Poloxamer P407/P188, PEG 8000	—	Temperature/genipin, HP-β-CD	Depression	Male ICR mice, male SD rats	Intranasally	Elevated levels of 5-HT and norepinephrine within the hippocampus and striatum; enhanced antidepressant-like effects	Qi et al. (2021)
Thermo-responsive		Alginate, poloxamer P407/P188	Sodium chloride	Temperature/Icariin	Depression	Male ICR mice, male SD rats	Intranasally	Fast-acting antidepressant effect; effective repair of neuronal damage in the hippocampus	Xu et al. (2020)
Thermo-responsive		Carboxymethyl chitosan	—	Temperature/PAOPA-loaded oxidized starch NPs	Schizophrenia	Male SD rats	Intranasally	Alleviation of negative symptoms like behavioral abnormalities associated with schizophrenia	Majcher et al. (2021)
Thermo-responsive		Poloxamers P407/P188	—	Temperature/Tetrandrine and HP-β-CD	Microwave-induced brain injury	Male Wistar rats	Intranasally	Improved spatial memory and spontaneous exploratory behavior	Zhang et al. (2020)
Thermo-responsive		Pluronic F127, PLGA–PEG-PLGA	—	Temperature/Salinomycin	Glioblastoma	Female BALB/c nude mice	Intratumorally	Superior drug release profile; reduced tumor growth	Norouzi et al. (2021)
Thermo-responsive		CS-HEC-HA, GP	—	Temperature/hUC-MSCs	Traumatic brain injury	Male SD rats	Intracerebrally	Enhanced survival and retention of MSCs; increased neuron survival; improved learning and memory abilities	Yao et al. (2019)
Thermo-responsive		Chitosan, poloxamer P408	—	Temperature/Curcumin-loaded mesoporous silica NPs	Alzheimer’s disease	Female Swiss albino mice	Intranasally	High permeation across nasal mucosa; reverting the cognitive deficit	Ribeiro et al. (2022)
Thermo-responsive		Pluronic F127, SCMC	—	Temperature/Oxcarbazepine-loaded chitosan NPs	Epilepsy	Male SD rats	Intranasally	Increased anti-inflammation; decreased seizure score and prolonged survival	Abou-Taleb and El-Ganainy, (2023)
Enzyme-responsive		Para-sulfobenzoic acid	—	MMP-9/peptide SFNV	Traumatic brain injury	Female C57BL/6J mice	Intracerebrally	Providing ECM like environment with sulfate functionalities; supporting the survival of neurons	Adak et al. (2020)
Enzyme-responsive		Hyaluronic acid, chitosan, heparin sulfate	Adipic acid dihydrazide	MMP/SDF-1α- and bFGF-loaded polyelectrolyte complex NPs	Ischemic stroke	Male SD rats	Intracerebrally	Enhanced neurogenesis and angiogenesis; facilitated recovery of neurological behavior	Jian et al. (2018)
Enzyme-responsive		Triglycerol monostearate	—	MMP/TMZ, O6-benzylamine	Glioma	Male BALB/c nude mice	Intratumorally	Enhanced effectiveness of TMZ in suppressing glioma growth; decreased recurrence rate of TMZ-resistant glioma following surgical intervention	Zhao et al. (2020)
Enzyme-responsive		PEG-b-PTyr-b-PAsp	—	Proteinase K/volasertib, ANG-CPP	Glioblastoma	Female BALB/c nude mice	Intravenously	Reduced toxicity; prolonged circulation time; better anti-tumor activity	Fan et al. (2021)
Enzyme-responsive		PEG-bis-AA	UV light	Hyaluronidase and esterase/HA-DXM	Traumatic brain injury	Male SD rats	Intracerebrally	Sustained release of dexamethasone; higher neuronal cell survival; improved motor functional recovery	Jeong et al. (2021)
Photo-responsive		GelMA-imid	Blue light	Blue light/PDA@SDF-1α NPs, hASMCs	Traumatic brain injury	Male SD rats	Intracerebrally	Promoting the migration of hAMSCs to injury site; promoting the differentiation of hAMSCs into nerve cells	Zheng et al. (2021)
Electro-responsive		Chitosan, aniline pentamer, carboxylated Pluronic F127	Pluronic	Electricity/VEGF	Hippocampus ischemia	Adult male Wistar rats	Intracerebrally	Decreased infarction volume; improved hippocampal dependent learning and memory performance	Nourbakhsh et al. (2020)
ROS-responsive		HA-PBA; PVA	—	ROS/Desferoxamine mesylate	Traumatic brain injury	Male SD rats	Intracerebrally	Reducing iron overload; diminishing ROS level; promoting neuronal recovery after trauma	Qiu et al. (2024)
Hypoxia-responsive		Poly(phosphorylcholine)	Azobenzene	Hypoxia/doxorubicin hydrochloride	Glioblastoma	Nude female BALB/c mice	Intravenously	Prolonged blood circulation and favorable immune compatibility; effective penetration through the BBB; favorable tumor inhibition effect	Peng et al. (2021)
Dual-responsive		GelMA, PPS60	Blue Light	ROS, MMP/procyanidins	Traumatic brain injury	Male ICR mice	Intracerebrally	Inhibiting the oxidative stress response by depleting ROS	Huang et al. (2022)
Dual-responsive		HA-PBA, PVA	—	Glucose, ROS/NSC-EVs	Stroke	Type 2 diabetes mellitus mice	Intracerebrally	Excellent angiogenic effect; improved neurobehavioral recovery	Jiang et al. (2022)
Dual-responsive		Deacetylated gellan gum, poloxamer P407, sodium alginate	–	Temperature, ion/timosaponin BII, HP-β-CD, chlorobutanol	Alzheimer’s disease	C57BL/6J mice	Intranasally	Decreased levels of proinflammatory mediators; enhanced memory and language functions; reduced cognitive decline	Chen et al. (2020)
Triple-responsive		Poloxamer P188/P407	—	Temperature, pH, ROS/Olz/RDPA NPs	Depression	Male Wistar rats	Intranasally	Efficient delivery of NPs to the brain; combined ROS depletion and inhibition of 5-HT dysfunction; alleviation of depression-like behaviors	Liu et al. (2023)
Dual-responsive		Poly (propylene sulfide)120	Triglycerol monostearate	ROS, MMP/curcumin	Traumatic brain injury	Male ICR mice	Intracerebrally	ROS depletion; reduced inflammation and brain edema; improved neural regeneration and behavior recovery	Qian et al. (2021)
Dual-responsive		Gelatin, carboxylic acid-terminated oligosulfamethazine	—	Temperature, pH/paclitaxel	Glioblastoma	BALB/c mice	Intratumorally	Sustained release of paclitaxel; inhibition of tumor growth	Kang et al. (2021)
Dual-responsive		Chitosan-g-poly(N-isopropylacrylamide)	Aldehyde-terminated difunctional polyurethane	Temperature, pH/SLP2 shRNA, (GO-CET)/CPT11	Glioblastoma	Female BALB/c nude mice	Intratumorally	60% tumor size reduction	Lu et al. (2020)

5-HT: 5-hydroxytryptamine; ANG-CPP: angiopep-2-docked chimeric polypeptide polymersome; BBB: blood-brain barrier; bFGF: basic fibroblast growth factor; CS-HEC-HA: chitosan, hydroxyethyl cellulose and hyaluronic acid; ECM: extracellular matrix; GelMA: gelatin methacrylate; GelMA-imid: imidazole groups-modified gelatin methacrylate; (GO-CET)/CPT11: cetuximab-conjugated graphene oxide; GP: β-glycerophosphate; HA-PBA: phenylboronic acid grafted hyaluronic acid; hAMSCs: human amniotic mesenchymal stromal cells; HP-β-CD: hydroxypropyl-β-cyclodextrin; HA-DXM: dexamethasone-conjugated hyaluronic acid; hUC-MSCs: human umbilical cord mesenchymal stem cells; MMP: matrix metalloproteinase; NPs: nanoparticles; NSC-EVs: neural stem cell-derived extracellular vesicles; Olz/RDPA: olanzapine/hexa-arginine-conjugated dextran coupled phenylboronic acid pinacol ester; PDA: polydopamine; PEG: polyethylene glycol; PEG-b-PTyr-b-PAsp: poly(ethylene glycol)-b-poly(L-tyrosine)-b-poly(Laspartic acid); PEG-bis-AA: poly (ethylene) glycol-bis-(acryloyloxy acetate); PLGA–PEG-PLGA: poly (dl-lactide-co-glycolide-b–ethylene glycol-b-dl-lactide-co-glycolide); PPS60: poly(propylene sulfide)60; PVA: polyvinyl alcohol; SCMC: sodium carboxymethyl cellulose; ROS: reactive oxygen species; SD: Sprague-Dawley; SDF-1α: stromal-cell-derived factor-1α; shRNA: short hairpin RNA; SLP2: stomatin-like protein 2; TMZ: temozolomide; UV: ultraviolet; VEGF: vascular endothelial growth factor.

## 3 Design principle of stimuli-responsive hydrogels for brain disease treatment

Given the intricate composition of brain tissue, the restoration of functional connectivity between axons, neural circuits, and non-neuronal cells poses a significant challenge for stimuli-responsive hydrogels in treating brain diseases (Halim et al., 2021). These hydrogels must exhibit excellent biocompatibility and biodegradability to minimize immune activation during treatment (Lee et al., 2021; Zamproni et al., 2021). Additionally, the mechanical properties of the hydrogels must closely mimic the softness of brain tissue, typically ranging from 0.1 to 0.3 kPa, to favor neural growth, migration, and neurite extension (Xia et al., 2017; Yang et al., 2017). Substrate topography also plays a pivotal role, providing nano- or micro-structured environments that can guide cell growth and regulate neural cell differentiation (Seo et al., 2018; Wang et al., 2019). Moreover, porosity is a critical factor that influences nutrient diffusion, waste removal, and cell seeding, penetration, and growth within the hydrogel matrix (Bružauskaitė et al., 2016; Shi et al., 2022). Typically, pore sizes in the range of 95–150 µm are considered optimal for neural tissue culture (Shi et al., 2022). To support cell attachment and enhance cellular interactions, immobilization of substances such as poly-L-lysin, fibronectin, gelatin, laminin, collagen, and peptides (RGD, IKVAV, GRGDS, mi-GDPGYIGSR, and mi-GQASSIKVA) is often required (Balion et al., 2020; Long et al., 2020; El-Husseiny et al., 2022). Last, conductive materials show promise in electrical stimulation treatment and recording, but their safety and biocompatibility must be rigorously examined due to concerns regarding cytotoxicity and chronic inflammation (Khan et al., 2022).

Therapeutic agents, including small compounds, peptides, proteins, nucleic acids, cells, and extracellular vesicles, are the key component of stimuli-responsive hydrogels, playing a pivotal role in brain disease treatment by reducing ROS damage and inflammation, promoting neural regeneration, and inducing tumor cell death (Ghosh et al., 2024). To achieve precise and controlled targeted delivery of therapeutic agents, these hydrogels capitalize on the patient’s physiological and pathological environments. Thermo-, ion strength-, and pH-responsive hydrogels exploit temperature variations, ion particles, and pH levels in nasal cavities or brain tissues to form *in situ* gels. Elevated ROS levels and enzyme expression in diseased brain tissues enable ROS- or enzyme-responsive hydrogels to release therapeutic agents with precision. In diabetics, high glucose triggers glucose-responsive hydrogels to release therapeutic agents. These hydrogels revolutionize brain disease treatment, providing controlled and targeted drug delivery.

Recently, hydrogels with self-assembly, self-healing, nanocomposite hybrid, and nano-size properties show promise for brain disease treatment (Jooken et al., 2023). Peptide-based self-assembling hydrogels exhibit high water content, tunable properties, and injectability, of which self-assembly process is governed by precise hydrophobic/hydrophilic interactions and hydrogen bond formation (Oliveira et al., 2022). Self-healing hydrogels overcome strength limitations, with reversible polymer chains enabling spontaneous repair and enhanced durability, in which chitosan and alginate are commonly used in their production (Zhu et al., 2023). Nanocomposite hydrogels hold superior physical, electrical, and biological properties, particularly for neural regeneration (Wang et al., 2022). Nano-sized hydrogels, or nanogels, are 20–200 nm in size, offering superior targeting and tissue access (Hajebi et al., 2019; Peng et al., 2021).

## 4 Preclinical progress in utilizing stimuli-responsive hydrogels for brain disease treatment

Brain diseases typically exhibit pathological features such as neural loss or death, vascular dysfunction, inflammation, oxidative stress, and increased expression of MMPs in affected tissues (El-Husseiny et al., 2022; Ghosh et al., 2024). These environmental cues can be exploited by hydrogels that respond to various stimuli, enabling targeted therapeutic delivery. Additionally, therapeutic agents like neuroprotective drugs, peptides, antioxidants, and growth factors can be released by hydrogels to alleviate these pathological conditions. Exceptionally, hypoxia within GBM tissues can be targeted by hypoxia-responsive hydrogels loaded with antitumor drugs, ranging from small compounds to nucleic acids. Notably, nanocomposite hybrid hydrogels and nanogels demonstrate remarkable performance in drug delivery, effectively traversing BBB. Recent preclinical *in vivo* studies evaluating the therapeutic efficacy of stimuli-responsive hydrogels in brain disease treatment are listed in Table 1.

### 4.1 Bain injury

#### 4.1.1 Traumatic brain injury

Encapsulating mesenchymal stromal cells (MSCs) or growth factors into hydrogels can enhance anti-inflammatory effects and promote neural regeneration. Yao et al. developed an injectable thermo-responsive hydrogel using chitosan, hydroxyethyl cellulose, hyaluronic acid, and beta-glycerophosphate (Yao et al., 2019). This hydrogel mimics brain tissue’s rheological behavior, liquefying at <25°C and solidifying at body temperature. When loaded with human umbilical cord-derived MSCs (hUC-MSCs) and injected into TBI rat brains, it enhanced MSC survival and retention, resulting in elevated brain-derived neurotrophic factor, neuron survival, and improved learning and memory, outperforming that of MSC-alone treatment. Separately, Zheng et al. created a blue light crosslinked hydrogel with imidazole-modified gelatin methacrylate and polydopamine/stromal-cell derived factor-1 (PDA@SDF-1α) nanoparticles loaded with human amniotic MSCs (hAMSCs). This hydrogel promoted hAMSC migration to injury sites and neuronal differentiation, repairing cryogenic brain injury in rats (Zheng et al., 2021).

Utilizing enzymes in the injured brain tissues, Adak et al. designed MMP9-responsive peptide-based hydrogels (SFNV) for TBI treatment. These hydrogels released neuroprotective peptides (NAVSIQ) by MMP9-mediated cleavage of PLGL tetrapeptide linker, and promoted neurogenesis in hippocampal regions of cryogenic injury mice (Adak et al., 2020). In another case, Jeong et al. conjugated dexamethasone with hyaluronic acid in a poly (ethylene) glycol-bis-(acryloyloxy acetate) hydrogel (PEG-bis-AA), This formulation sustained dexamethasone release, reducing inflammatory cytokines and enhancing motor recovery in TBI rats 7 days post-injury (Jeong et al., 2021).

Iron overload worsens neurodegeneration by promoting ROS production (Tang et al., 2020). Qiu et al. incorporated desferoxamine mesylate, an iron chelator, into a boron ester-bonded hydrogel composed of 3-aminophenylboronic acid-grafted hyaluronic acid and polyvinyl alcohol (PVA). This hydrogel self-healed and responded to ROS due to the boron ester bond, alleviating iron overload and oxidative stress in brain-injured rats, improving motor, learning, and memory functions (Qiu et al., 2024). For better drug delivery and release, Qian et al. created an injectable dual ROS/enzyme-responsive hydrogel composed of poly (propylene sulfide)120 (PPS120) and curcumin within a triglycerol monostearate (TM) hydrogel. Injection into TBI mice brains caused MMPs to cleave the TM coat, PPS120 to react with ROS, and curcumin to scavenge ROS. This reduced reactive glia cells, inflammation, brain edema, and improved BBB integrity, enhancing nerve regeneration and behavioral recovery in TBI mice (Qian et al., 2021).

#### 4.1.2 Stroke

Antioxidants and growth factors are embedded in hydrogels to promote neurogenesis and angiogenesis. In a case, a chitosan micellar hydrogel encapsulated hydrophilic minocycline and hydrophobic edaravone drugs for stroke treatment. Injection in rats promptly released the hydrophilic drug and sustains release of the hydrophobic drug, leading to significant behavioral recovery (∼84%) due to sequential anti-inflammatory and neurogenesis effects (Lin et al., 2023). Another study reported the functional repair of the hippocampus post-ischemia using a pluronic-chitosan/aniline-pentamer hydrogel loaded with vascular endothelial growth factor (VEGF). This hydrogel mimics brain tissue conductivity (10^–4^ S/cm), enabling sustained VEGF release upon intracerebral administration. This approach significantly reduced infarct size by >70% and improved hippocampal-dependent learning and memory, outperforming VEGF delivery alone (Nourbakhsh et al., 2020). A third study used polyelectrolyte complex nanoparticles loaded with SDF-1α and basic fibroblast growth factor, modified with MMP-cleavable peptides, and combined with hyaluronic acid to form enzyme-responsive hydrogels. These hydrogels exhibited superior neurological recovery compared to free growth factors or bare hydrogels in an ischemic stroke model through intracerebral administration, enhancing neurogenesis and angiogenesis (Jian et al., 2018).

ROS and high blood glucose hamper stroke recovery. Jiang et al. designed a dual glucose/ROS-responsive hydrogel loaded with neural stem cell-derived extracellular vesicles (NSC-EVs) for diabetic stroke treatment (Jiang et al., 2022). The hydrogel, made from crosslinking PBA-modified hyaluronic acid with PVA, prolonged EV retention and activity in the brain. NSC-EVs released miRNAs vital for angiogenesis, reducing brain atrophy and enhancing neurobehavioral recovery in diabetic stroke mice.

### 4.2 Neurodegenerative diseases

The use of intranasal administration has demonstrated significantly improved drug delivery for the treatment of Alzheimer’s disease. For instance, Curcumin-loaded mesoporous silica nanoparticles in chitosan and P407 hydrogel improved permeation of drugs and cognitive function in an Alzheimer’s mouse model (Ribeiro et al., 2022). In another study, Chen et al. developed dual pH/thermo-responsive hydrogels with neuroprotective timosaponin BII for Alzheimer’s disease (Chen et al., 2020). This hydrogel integrated ion-sensitive deacetylated gellan gum, thermo-sensitive Poloxamer 407, and sodium alginate, harnessing rapid sol-gel transition triggered by heat and Ca^2+^ in the nasal cavity. Studies on mice showed improved memory, language, reduced cognitive decline and neuroinflammation.

In recent 5 years, there has been a lack of preclinical animal studies for the treatment of Parkinson’s disease.

### 4.3 Psychiatric disorders

#### 4.3.1 Major depressive disorder

Intranasal administration of thermo-responsive gels can effectively deliver drugs to brain tissue. An antidepressant drug, genipin, was combined with hydroxypropyl-β-cyclodextrin (HP-β-CD) and mixed with P407/P188/PEG8000 to create a thermo-responsive hydrogel for intranasal delivery. This hydrogel effectively and sustainably released genipin in mouse brains, improving antidepressant effects (Qi et al., 2021). In another case, icariin was encapsulated in alginate nanogels and integrated into a P407/P188 hydrogel for sustained release after intranasal administration, leading to rapid antidepressant effects in mice and rat models (Xu et al., 2020). Depression is linked to oxidative stress, making antioxidant therapy promising (Bhatt et al., 2020). Recently, a combination of nanoparticles and hydrogels demonstrated impressive drug delivery capabilities (Liu et al., 2023). In this system, amphiphilic polymers (DEX-g-PBAP) made of phenylboronic acid pinacol ester (PBAP) and dextran (DEX) with ROS-sensitive borate ester bonds were used to load antidepressant olanzapine (Olz) to form nanoparticles (Olz/DP NPs). Olz/DP NPs were then modified with amino borane to get Olz/DPA NPs, and further conjugated with hexa-arginine (R6) to create Olz/RDPA NPs. Olz/RDPA NPs were then encapsulated in a poloxamer (P407/P188) hydrogel. Upon intranasal administration in a rat model, the NPs were released from the hydrogel and transported to the brain via the nasal-brain pathway effectively. High ROS levels in the brain triggered NP breakdown, releasing Olz. Simultaneously, H_2_ released from amino borane scavenged •OH, reversing oxidative stress in the brain and alleviating depressive-like behaviors.

#### 4.3.2 Schizophrenia

PAOPA, a D2 allosteric modulator, significantly reduced schizophrenia-like symptoms in rats using hydrogels composed of oxidized starch nanoparticles and carboxymethyl chitosan. Its potency and bioavailability are emphasized by achieving relief with half the intraperitoneal dosage through intranasal delivery (Majcher et al., 2021).

#### 4.3.3 Epilepsy

Chitosan nanoparticles were employed to enhance the delivery of the antiepileptic drug Oxcarbazepine in Pluronic F127 (also known as poloxamer P407) and sodium carboxymethyl cellulose hydrogel (Abou-Taleb and El-Ganainy, 2023). This formulation exhibited improved antiepileptic activity and anti-inflammatory effects upon intranasal administration.

### 4.4 Brain tumors

Direct intratumoral injection of stimuli-responsive hydrogels that leverage tumor microenvironment factors, such as low pH and high enzyme expression, is an efficient strategy to overcome the BBB impediment for drug delivery. Recently, injectable thermo-responsive hydrogels encapsulating salinomycin using copolymers Pluronic F127 and poly (dl-lactide-co-glycolide-b–ethylene glycol-b-dl-lactide-co-glycolide) (PLGA–PEG-PLGA) improved drug release and cytotoxicity, significantly reducing GBM tumor growth, surpassing the effect of free salinomycin alone (4-fold) (Norouzi et al., 2021). In another case, a dual pH/thermo-responsive hydrogel, made from carboxylic acid-terminated oligosulfamethazine and gelatin, transforms rapidly sol-to-gel in response to body temperature and low pH. This hydrogel can sustainably release paclitaxel intratumorally thanks to gelatin’s MMP-cleavage site for antitumor effects in GBM mice (Kang et al., 2021). In a third study, TM-based MMP9-responsive hydrogels loaded with Temozolomide and O6-benzylamine prevented recurrence in post-operative glioma models (Zhao et al., 2020). Encapsulation of therapeutic nucleic acids is also a promising method for treating GBM. Recently, an injectable drug/gene delivery system using a thermosensitive chitosan-based polymer solution to entrap stomatin-like protein 2 (SLP2) shRNA and irinotecan (CPT-11)-loaded cetuximab (CET)-conjugated graphene oxide (GO-CET/CPT11) has been created (CPN@GO-CET/CPT11@shRNA). This formed a hydrogel depot for localized, sustained delivery of therapeutics. Efficient transfection of U87 cancer cells with SLP2 shRNA was achieved using this hydrogel, demonstrated *in vivo* using tumor-bearing mice. This hydrogel offers extended drug release and shRNA delivery advantages, broadening GBM treatment modalities (Lu et al., 2020).

Nanocomposite hybrids and nanosized gels, as emerging nanotechnologies, have demonstrated significant potential in enhancing intravenous drug delivery for the treatment of GBM. Plk1 upregulation in GBM can be suppressed by Plk1 inhibitor Volasertib. Loaded into angiopep-2-decorated chimeric polypeptide polymersome, Volasertib forms a nanogel with a size of approximately 76 nm from poly(ethylene glycol)-b-poly(L-tyrosine)-b-poly(Laspartic acid). This nanogel traversed BBB and GBM membranes via lipoprotein receptor-related protein 1 (LRP-1)-mediated transcytosis and endocytosis respectively, rapid releasing of Volasertib by proteinase K, suppressing GBM growth and enhancing survival in mice (Fan et al., 2021). In another case, hypoxia-degradable zwitterionic phosphorylcholine nanogel, ^H^PMPC, penetrated the BBB by mimicking cell membrane structure after intravenous administration, releasing doxorubicin by azobenzene moiety degradation in tumor tissues of GBM mouse models (Peng et al., 2021).

## 5 Discussion

**FIGURE 1:**
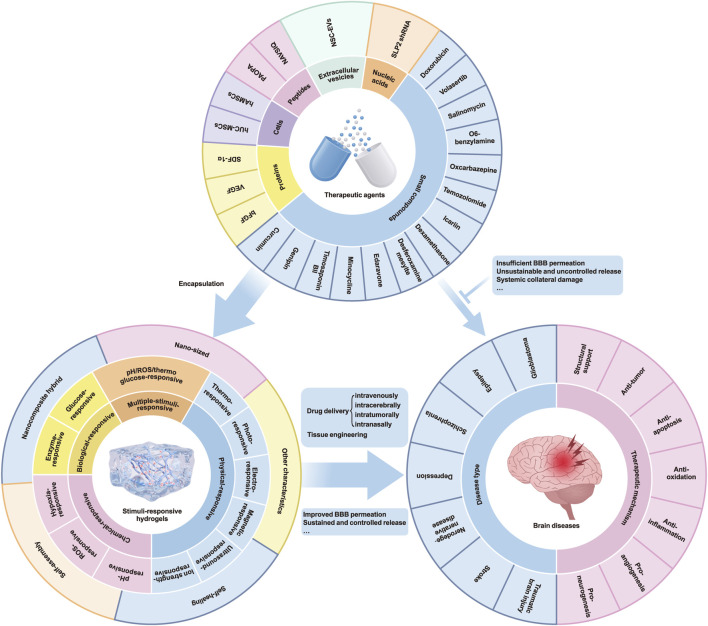
Schematic diagram of stimuli-responsive hydrogels for brain disease treatment in recent preclinical *in vivo* study. BBB: blood-brain barrier; bFGF: basic fibroblast growth factor; hAMSCs: human amniotic mesenchymal stromal cells; hUC-MSCs: human umbilical cord mesenchymal stem cells; NSC-EVs: neural stem cell-derived extracellular vesicles; ROS: reactive oxygen species; SDF-1α: stromal-cell-derived factor-1α; SLP2 shRNA: stomatin-like protein 2 short hairpin RNA; VEGF: vascular endothelial growth factor.

In this review, we examined the distinguishing features of a range of stimuli-responsive hydrogels, particularly those that respond to thermal, photonic, magnetic, electrical, and biological cues. Our emphasis lies in highlighting how these hydrogels can capitalize on the pathophysiological features of diseased brains by developing responsive biomaterials. Additionally, we outlined their varied applications and underlying therapeutic mechanisms, including neurogenesis promotion, anti-inflammatory, anti-apoptotic, anti-oxidative, and angiogenic effects, all contributing to the enhancement of therapeutic outcomes (summarized in Figure 1). These hydrogels offer numerous benefits, including precise targeting, controlled release, mechanical support, and biocompatibility, rendering them promising candidates for the treatment of brain diseases in the future.

These advanced systems, despite their potential, exhibit several limitations that necessitate careful consideration during their application based on the specific pathophysiological characteristics of the target disease. For instance, stimuli-responsive systems relying on alterations in pH, ROS, or enzymes may exhibit suboptimal sensitivity to nuanced microenvironmental changes, potentially resulting in underperformance. Additionally, the *in vivo* microenvironment poses challenges, such as the formation of a protein corona around nanoparticles, which can significantly reduce or even eliminate the therapeutic efficacy of these systems. Other inherent limitations include thermal denaturation, UV-induced carcinogenesis, insufficient mechanical strength, and material toxicity. Furthermore, the hydrogels’ propensity for high swelling can result in elevated local tissue pressure, which may compromise their mechanical integrity and exacerbate secondary brain injury. Therefore, a thorough understanding of these limitations and careful evaluation of their applicability in specific cases are essential for the optimal utilization of these systems in therapeutic applications.

The integration of nanotechnology, artificial intelligence, and three-dimensional printing into the development of hydrogels presents a compelling opportunity for the creation of innovative therapeutic strategies in brain diseases (Gao et al., 2023). Yet, a rigorous preclinical evaluation encompassing safety, efficacy, and stability is paramount before clinical translation. Key challenges include elucidating hydrogel-neural microenvironment interactions, minimizing immunogenicity, and optimizing targeted delivery. While animal models offer insights, they may not fully recapitulate human disease complexities. Therefore, bridging the gap between *in vitro*/*in vivo* models and human diseases remains a significant challenge (Shabani et al., 2023). Future research must prioritize enhancing hydrogel biocompatibility, especially by exploring natural materials that facilitate cellular recognition and integration. Identifying the optimal therapeutic window in various brain diseases, considering disease progression, is crucial for maximizing therapeutic potential. Additionally, scalable manufacturing, regulatory adherence, and economic viability are essential for successful commercialization.

Collectively, future trends in stimuli-responsive hydrogel development emphasize: multifunctional biomaterials integrating therapy, imaging, tissue regeneration, stem cell support, immune modulation, and antibacterial properties; exploration of novel stimuli like inflammatory enzymes for enhanced targeting; optimization of biomaterials for biosafety, efficacy, and cost-effectiveness; addressing patient compliance, convenience, and cost issues; and advancing our pathological understanding of brain diseases to design effective therapies. Collaborations across disciplines are key to translating hydrogel technologies to clinical applications.

In conclusion, stimuli-responsive hydrogels hold significant potential for revolutionizing the treatment of brain diseases. A multidisciplinary approach that integrates expertise from various fields is crucial for advancing this research and ultimately bringing new therapeutic options to patients.
